# Rapid Microwave-Assisted Cisplatin-Loaded Solid Lipid Nanoparticles: Synthesis, Characterization and Anticancer Study

**DOI:** 10.3390/nano10030510

**Published:** 2020-03-11

**Authors:** Hibah M. Aldawsari, Sima Singh

**Affiliations:** 1 Department of Pharmaceutics, Faculty of Pharmacy, King Abdulaziz University, Jeddah 21589, Saudi Arabia; 2 Discipline of Pharmaceutical Sciences, College of Health Sciences, University of KwaZulu Natal, Durban 4000, South Africa; simasingh87@gmail.com

**Keywords:** stearic acid, cisplatin, microwave-assisted technique, breast cancer, solid lipid nanoparticles

## Abstract

Cisplatin is one of the most leading potent chemotherapy drugs prescribed for the treatment of most solid tumors. However, the induction of toxicities and the development of resistance restricts its applications. Efforts are made in the proposed study to control the delivery of cisplatin to tumor sites by incorporating it into solid lipid nanoparticle (SLNs) drug carriers. By considering this fact, in the current research work, a single-step, one-pot, microwave-assisted technology was used to produce cisplatin-loaded SLNs. The shape of the SLNs was observed to be spherical, with a uniform size distribution of 74.85 nm, polydispersity index (PDI) of 0.311, and zeta potential of −20.8 mV. The percentage of encapsulation efficiency was found to be 71.85%. *In vitro* drug release study was calculated to be 80% in 24 h. The formulation in blood was found to be safe; a study of hemolysis confirmed this. Breast cancer cell line MCF-7 was used to test cytotoxicity and cellular interaction of cisplatin-loaded SLNs with an IC_50_ value of 6.51 ± 0.39 μg/mL. Overall, the results of our findings show that the approach of SLNs-based, cisplatin-based, drug delivery has led to increased sustainability in breast cancer therapy with superior biocompatibility.

## 1. Introduction

Breast cancer (BC) in women throughout the globe is one of the deadliest and secondary-most prevailing causes of mortality. It is expected and anticipated to surpass the mortality rate of heart diseases in upcoming years [1]. The American Cancer Society (ACS) estimates that 29 percent of the incidence and 15 percent of breast cancer mortality are the global values [2]. Clinically, BC is a heterogeneous disease condition with multifactorial etiology. There is still unclear etiology of breast cancer. A blend of risk factors remain the foremost accountable factors for breast cancer, which include genetic, lifestyle, nutritional, environmental, hormonal alterations, and exposure to ionizing radiation [3,4,5,6]. Molecular biology of breast cancer is challenging because so many factors lead to breast cancer progressions, such as breast cancer gene (BRCA) [7], BRCA2 [8], p53 gene mutations [9], and crosstalk between different signaling pathways [10]. Cell signaling pathways facilitate normal proliferation, transcription, development, migration, differentiation, and death programs in healthy cells [11]. However, in the case of breast cancer cells, these ordinary programs are reversed. Various signaling pathways play a significant role in the progression and the advancement of breast cancer. It is mainly initiated by interactions between growth factors and their receptors—mainly human epidermal growth factor receptors (HER-2) [12], vascular endothelium growth factor (VEGF) and their ligands [13], as well as insulin-like growth factor (IGF) and insulin-like growth factor 1 receptors (IGF-1R) [14].

Treating breast cancer is a massive clinical hurdle due to its heterogeneity, complexity, and aggressiveness [15]. Conventional treatment methods recommended for BC typically include operation, chemotherapy, radiotherapy, and hormonal treatment therapy [16]. Despite outstanding medical science, the long-term rate of survival in the advanced stage of BC is five years [17]. Although these medications are incredibly efficient, they have some undesirable severe side effects that restrict further applications. Previously, researchers have suggested that novel target-based treatment is a good alternative. Management of BC involves various treatment options, such as platinum and taxanes, which have limited effectiveness in metastatic breast cancer [18]. Cisplatin has widely transformed cancer management and prevails as a widely prescribed therapeutic [19]. However, cisplatin treatment presents significant constraints due to drug dose-induced systemic toxicities, such as neurotoxicity, ototoxicity, gastrointestinal disorders, myelosuppression, and nephrotoxicity [20]. Moreover, more than 90% of the drugs were reported to be flushed instantly by glomerular filtration, which led to therapeutic ineffectiveness for patients [21]. Sadly, most of the convenient treatment choices that depend on cisplatin do not efficiently target breast cancer [22]. Despite cisplatin being effective in the treatment of BC, dose-dependent toxicities have led to discontinuation of therapy due to patient complications. To provide an impactful, feasible solution to overcome the disadvantages of existing cisplatin-based treatment, an ideal system needs to be developed that can target drugs to specific body sites and monitor their release for more extended periods. Developing a drug delivery system that can boost cisplatin concentration in the tumor while decreasing it in the kidney would provide a successful strategy for improving antitumor efficacy and reducing nephrotoxicity in the clinical use of cisplatin. It is anticipated that site-specific delivery of drugs to the breast site will help in minimizing the side effects by enhancing the effectiveness of the given therapy [23].

Several site-specific drug delivery-based formulations using different nanocarrier systems like liposomes, dextran conjugates, as well as polymeric micelles, have been reported to improve the effective release of cisplatin and to govern its premature release [24]. In the last few decades, lipid-based carrier systems have been attractive to researchers. In the literature, two major types of lipid-based nanoparticles have been reported, i.e., nanostructured lipid carriers (NLCs) and solid lipid nanoparticles (SLNs). Out of these, existing traditional colloidal carrier systems, including emulsions, liposomes, and polymeric micro and nanoparticles, have some major drawbacks, such as short lifespan, poor durability, poor encapsulation effectiveness, very quick removal by the reticuloendothelial system (RES), cell interactions or adsorption, and intermembrane transition [25,26,27]. Solid lipid nanoparticles (SLNs) have been developed as an alternative lipid carrier system due to several advantages such as enhanced drug content, controllable drug-release profiles with targeting, and effective drug profiles with excellent physical stability [28,29]. Apart from these advantages, SLNs have technological benefits, such as their large-scale production, excellent retention flexibility, biodegradability, and biocompatibility that approve their safety and further consider their generally-recognized-as-safe (GRAS) status [30]. The present work is a follow-up on a study of previously reported work, which involved the preparation of cisplatin SLNs using a simple, one-pot, novel, microwave-assisted method. As compared to the previously reported conventional heating technique of the preparation of SLNs, microwave-assisted technology offers uniform microwave heating and yields lower polydispersity particles with the small size of particles. SLNs produced by the microwave-assisted technique reported to have more improved physicochemical characteristics as compared to the conventional method of preparation of SLNs [31]. The present study proposed to develop the cisplatin-loaded SLNs for the treatment of BC by a novel microwave-assisted technique. Outcomes of the results confirmed that encapsulated drug and *in vitro* drug release in cisplatin-loaded SLNs could facilitate site-specific drug delivery with improved local availability in a controlled drug-release pattern. We find a scalable approach that uses biocompatible excipients to deliver rapid synthesis of nanoparticles. This method offers a one-pot synthesis straight from the microwave reactor that yields purified nanoparticles.

## 2. Materials and Methods

### 2.1. Materials

Cisplatin, stearic acid, Tween 80 and glyceryl trimyristate were purchased from Sigma-Aldrich, Durban, South Africa. MTT (3-(4,5-dimethylthiazolyl-2)-2,5-diphenyltetrazolium bromide) was purchased from Sigma-Aldrich (St. Louis, MO, USA). All other chemicals and solvents used in the studies were either bought from Merck or Sigma-Aldrich, Durban, South Africa, and were of analytical grade. The pH study was performed with 0.1 M phosphate buffer which included sodium dihydrogen orthophosphate dehydrates (Merck, South Africa) and sodium dibasic dehydrates (Sigma-Aldrich, Taufkirchen, Germany). Throughout the experiment, double distilled water (DDW) was used.

### 2.2. Methods

#### 2.2.1. Selection of Lipids Based on Solubility Study

**Figure 1:**
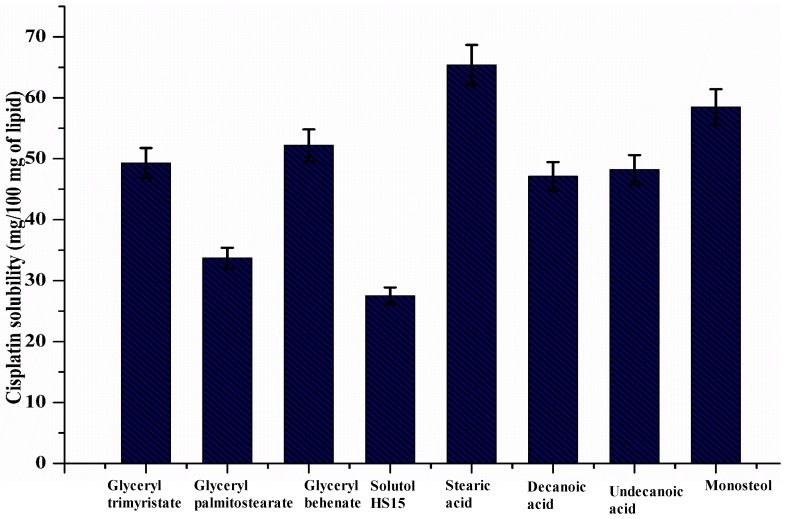
The solubility study of cisplatin in various lipids.

The solubility of cisplatin in different lipids was assessed with slight alterations according to previously reported techniques (*w/w* with respect to lipid mass) [31]. The more commonly used lipids were glyceryl trimyristate, glyceryl palmitostearate, glyceryl behenate, Solutol HS 15, stearic acid, decanoic acid, undecanoic acid, myristic acid, and Monosteol. The blend of lipid and cisplatin was melted at 80 °C and smeared on a glass slide to observe the presence or absence of insoluble drug crystals under a Leica microscope. Then, the physical mixture was completely dissolved in ethanol to determine the concentration of cisplatin using a UV-visible spectrophotometer (UV-1800, Shimadzu, Kyoto, Japan) at 307 nm [32]. The measurements of the solubility were performed in triplicate. The graph of the solubility studies is represented in Figure 1. Based on the maximum solubility of cisplatin in lipid, we selected lipid for the preparation of SLNs. Results showed that cisplatin has more solubility affinity in stearic acid compared to other lipids. Hence, we selected stearic acid for further preparation of cisplatin-loaded SLNs.

#### 2.2.2. Preparation of Cisplatin-Loaded SLNs: Microwave-Assisted Technique

A microwave-based technique was used to prepare cisplatin-loaded SLNs with slight modifications to the method reported earlier [31]. A fixed amount of cisplatin (120 mg) was mixed with stearic acid (100–300 mg), Tween 80 (50 mg) or Solutol HS 15 (50 mg) and water (1.2 mL) followed by heating at 90 °C in a microwave reactor tube under constant stirring. The tube reactor containing the mixture was run at 90 °C using a 2.45 GHz microwave synthesizer at maximum power (300 W) (Discover LabMate, CEM Corp., Matthews, NC, USA), and constituted a single one-pot synthesis of the microemulsion (oil in water type). The reaction mixture temperature (90 °C) was maintained for 10 min at 10 W variable microwave power. Finally, the prepared hot microemulsion (1.2 mL) was rapidly dispersed into 30 mL of cold water (2–8 °C) containing PF68 (75–400 mg), ascorbic acid (10 mg) and Tetraxetan (2 mg) under stable stirring (400 rpm) to produce SLNs. The final nanodispersion (30 mL) was lyophilized to get a solid powder, which was further reconstituted in 1.2 mL of 5% dextrose solution for subcutaneous delivery. Each formulation (F1–F10) was prepared using the same procedure in the desired ratio for a given period of time to obtain the homogenous formulation. Each formulation was reconstituted and subjected to further evaluations. Out of these ten formulations, only stearic acid-based SLNs showed higher cisplatin solubility and stability (after redispersion in 5% dextrose solution) compared to other formulations. Table 1 represents the optimization excipients used in preparation of cisplatin-loaded SLNs. Formulation code F8 was further selected for further characterization and evaluation study.

#### 2.2.3. Characterization of Nanoparticles

##### Morphological Evaluation

The shape and size of the optimized finally prepared SLNs were investigated by transmission electron microscopy (TEM) on a Jeol, JEM-1010 (Kitakyushu, Japan). Morphology study of cisplatin-loaded SLNs was performed by TEM following the previously described procedure with some modifications [33,34]. In short, one drop of SLN suspension was positioned on carbon-coated copper grids. It was air-dried at room temperature before pictures were obtained. Images have been captured with the iTEM Soft Imaging Systems (SIS) Mega View III equipped with a side-mounted 3-megapixel digital camera.

##### Size, Polydispersity Index and Zeta Potential of SLNs

The photon correlation spectroscopy (PCS) technique was used to determine the size, size distribution, and zeta potential of the SLNs. Cisplatin SLNs was diluted in PBS for this purpose, and its absorption was calculated at 630 nm using a spectrophotometry technique. Then, a Zetasizer tool (ZEN 3600, Malvern Instruments Ltd., Worcestershire, UK) was introduced to the suspension. 

##### Percent Entrapment Efficiency (% EE)

With a pre-established calibration curve, UV spectrophotometry (Shimadzu UV 1601, Japan) at 307 nm was used to determine the efficacy of encapsulation. In the phosphate-buffered solution (PBS, 0.01 M, pH 7.4), lyophilized nanoparticles were dissolved. It was sonicated for 20 min, and then centrifuged at 1000 rpm for 10 min. Two hundred microliters of the filtrate was taken off and diluted to 10 mL using the phosphate-buffered solution, and the amount of encapsulated drug was estimated using UV spectrophotometry. The equations of correlation and linearity (*r*^2^) were *y* = 0.0103*x* + 0.0056 and 0.9991, respectively. The percentage encapsulation efficiency (% EE) was calculated as per Equation (1):

(% EE) = (Total amount of VCM − the amount of VCM in supernatant)/Total amount of VCM × 100(1)

##### *In Vitro* Drug Release Measurement

Study of *in vitro* drug release was conducted by following the dialysis bag method. It was used to study drug release with some alteration of nanoparticulate systems. The release medium was the phosphate-buffered saline (PBS; 100 mM, pH 7.4). The dialysis bag was soaked in distilled water for 12 h before use (molecular weight cutoff: 12–14 kDa, Livingstone, NSW, Australia). In a dialysis bag, a 2 mL aliquot of prepared cisplatin-loaded SLNs was taken in amber-colored glass bottles, and 50 mL preheated release medium was immersed in the bottles. The bottles were placed in a 37 °C and 150 rpm thermostatic shaker. At predetermined time points, an aliquot of 5 mL of release medium was removed and immediately substituted with the same amount of new PBS to preserve sink conditions. Spectrophotometric analysis of the quantity of drug in the aliquot was performed at 307 nm.

##### *In Vitro* Hemolysis Activity

The hemo-biocompatibility of the newly synthesized cisplatin-loaded SLNs was carried out by blood hemolytic tests. It was carried out with a slide modification of the technique stated in the earlier protocol [35]. A blood sample of sheep (5 mL) was drawn from a covered bottle of ethylenediaminetetraacetic acid (EDTA) glazed tube and used within an hour. It was centrifuged at 1800 rpm for 10 min to distinguish red blood cells (RBCs) from blood samples. Separate RBCs were cleaned three times in order to behave as an adverse control with 5% suspension in PBS (pH 7.4) diluted with 0.9% saline solution. Different concentrations of cisplatin-loaded SLNs in the range of 0.0–500 μg/mL were treated with a 96-well plate 100 μL containing RBCs suspension. Eventually, the plate was stirred gently and further incubated for 3 h at 37 °C. The obtained supernatant was determined by a UV-vis spectrophotometer via a plate reader at 541 nm. The percentage of hemolysis was calculated using Equation (2):

Hemolysis (%) = (*At* − *Ac*)/(*Ax* − *Ac*) (2)

where *At* is the absorbance of treated supernatant, *Ac* is the absorbance of negative control, and Ax is the absorbance of positive control.

##### Cytotoxicity Assay

MCF-7 breast cancer cell lines were used to carry out the cytotoxicity study. Briefly, 96-well plates with 5000 cells/well of MCF-7 were taken, then, kept the cell lines to grow for 24 h. After completion of 24 h, in 96-well microtiter plates, 100 μL of cells were inoculated at plating densities depending on the developmental characteristics of each cell, and then were incubated at 37 °C for 24 h in a 5% CO_2_ incubator. After 24 h of cell incubation, cells were further treated with different concentrations of cisplatin-loaded SLNs, and were kept for 48 h at 37 °C. After 48 h, supernatant was removed and washed with 200 μL of phosphate-buffered saline (PBS). It was then substituted by addition of 200 μL of medium (DMEM for cancer cells) and 30 μL of 5 mg/mL of MTT. The plates were incubated at 37 °C for 4 h. The supernatant was removed from cell incubation and 50 μL of dimethyl sulfoxide (DMSO) was added. A microplate reader was used to evaluate MTT study at 570 nm wavelength and 630 nm reference wavelength. Experiments were performed in triplicate. IC_50_ values were determined from *in vitro* dose-response curves using linear regression analysis [36]. The data were analyzed using GraphPad Prism 5.0 (San Diego, CA, USA) and expressed as mean ± standard deviation. The percentage cell viability was calculated using Equation (3):

% Cell Viability = (A540 nm treated cells)/(A540 nm untreated cells) × 100(3)

## 3. Results and Discussions

### 3.1. Morphological Evaluation

**Figure 2:**
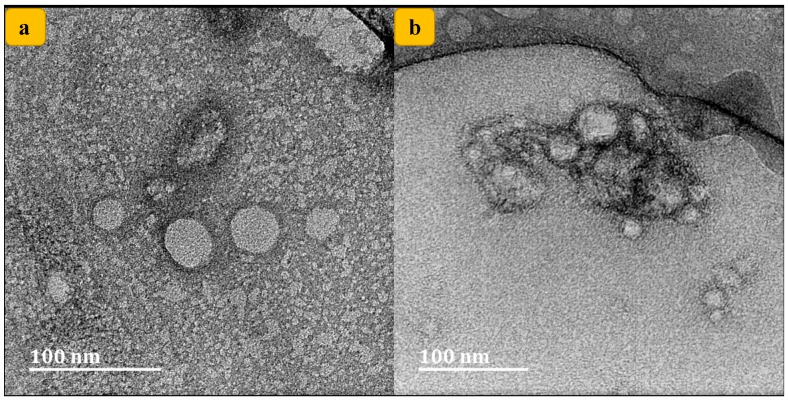
TEM images of cisplatin-loaded solid lipid nanoparticles (SLNs) with different magnification.

Using TEM, the cisplatin-loaded SLNs’ ultrastructural morphologies were examined. It was found that the shape of the prepared formulation was small, circular and homogenously uniform, displaying little aggregation, as shown in Figure 2a,b, similar to prior findings [37]. There was no significant morphological change in SLNs. In addition, the size acquired from the TEM study (about 100 nm) was further confirmed by dynamic light scattering (DLS) measurement, which was in the range of 70 to 100 nm. The lack of any uneven globular morphology indicates a full process of SLNs formation.

### 3.2. Size, Size Distribution, and Zeta Potential of SLNs

**Figure 3:**
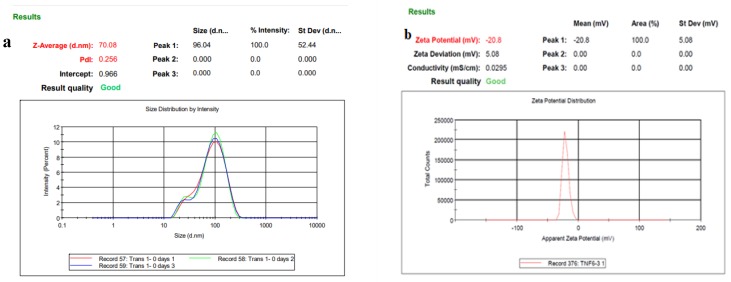
(**a**) DLS illustrating the size distribution of the particles formed; (**b**) Zeta potential of Cisplatin-loaded SLNs.

The intrinsic size, shape, and surface features need to be evaluated in the design and formulation of nanoscale delivery agents, as they all impact biocompatibility. This was done to accurately determine, in real time, the size, dispersion, and colloidal stability. Zeta (ζ) potential is the magnitude of the electrostatic potential produced between the particle and the dispersing medium at the edge of the slipping plane. Figure 3a,b shows that the size and ζ potential of each SLNs was found to be 70 nm in size with −20 (mv) zeta size. The polydispersity index (PDI) of the formulation was found to be 0.256. This ensures that the developed formulation was colloidally-stable dispersion of nanoparticles [38].

### 3.3. Percent Entrapment Efficiency (% EE)

Sedimentation through centrifugation is an easy, reliable, and accurate way to remove the unbound drug. Drug-bound nanoparticles will sediment at different velocities based on viscosity, size, and mass. Drugs bound to the nanoparticles are generally of higher size and mass and will consequently pellet, while unbound drug will remain in the supernatant. The percent encapsulation efficiency was calculated to be 71.85%, which correlated to TEM. The calculated encapsulation efficiencies were used to determine the actual drug content in nanoparticles based on the theoretical drug content from which dilutions were made for cytotoxicity studies.

### 3.4. In Vitro Drug Release Measurement

**Figure 4:**
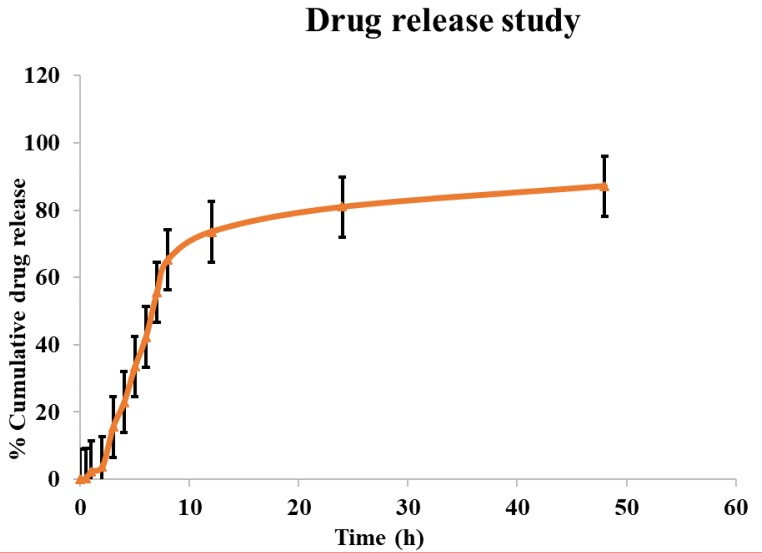
*In vitro* cumulative percentage drug release study of cisplatin-loaded SLNs for 48 h.

An *in vitro* drug release study was conducted for 48 h. The dialysis bag method was applied to calculate the amount of cisplatin produced from the SLNs. This is the most commonly used technique for estimating drug releases from SLNs reported in the literature. For the prepared formulations, a biphasic release profile was noted as shown by the first burst within 3 h followed by a prolonged release. Through two distinct processes, dissolution and diffusion, cisplatin loaded into the SLNs’ core leached gradually. Cisplatin’s initial burst release can be attributed to the drug’s rapid release incorporated into the shell. The drug release from the SLNs was anticipated to be very slow due to the strong core at body temperature. Figure 4 demonstrates the release profile of cisplatin prepared by methods supported by one-pot microwave. About 80% cisplatin was released within 12 h from the SLNs generated using the microwave-assisted novel method. These findings suggest that cisplatin incorporated in the SLNs is likely to remain associated with the nanoparticle.

### 3.5. In Vitro Hemolysis Activity

**Figure 5:**
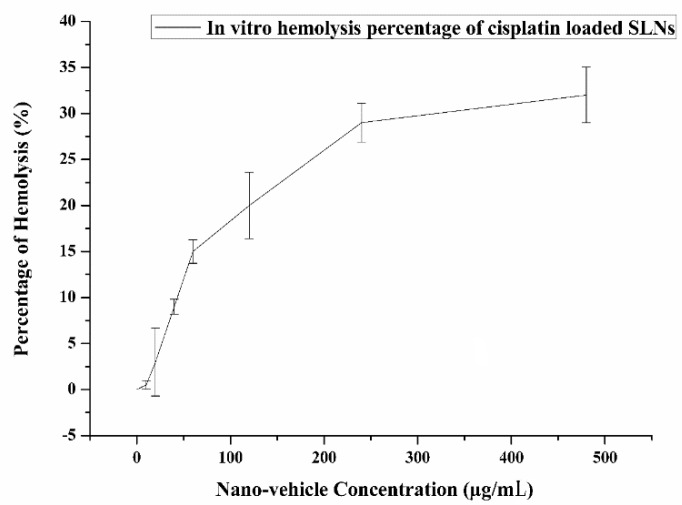
*In vitro* hemolysis percentage of cisplatin-loaded SLNs.

The hemolytic characteristics of cisplatin during chemotherapy are also accountable for the greater risk of blood disorders such as anemia. The assessment of SLNs hemo-compatibility should be regarded as one of the variables for evaluating systemic toxicity. The current findings show that cisplatin-loaded SLNs did not induce hemolysis. The current findings suggest that the percentage of hemolysis following cisplatin-loaded SLNs therapy was smaller relative to plain cisplatin therapy. The present research shows that there was no important difference in the proportion of hemolysis, suggesting that the formulations had no effect on circulating blood cells or on their production. These preliminary findings state that cisplatin loaded in SLNs can be used instead of free cisplatin for cancer treatment to minimize potential side effects. The *in vitro* hemolysis percentage of cisplatin-loaded SLNs is shown in Figure 5.

### 3.6. Cytotoxicity Assay

**Figure 6:**
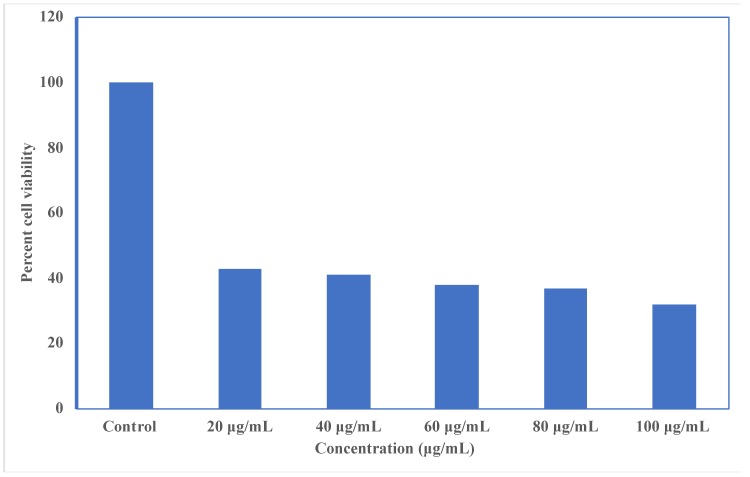
*In vitro* cytotoxicity and percent cell viability assay against MCF-7 breast cancer cell lines.

**Figure 7:**
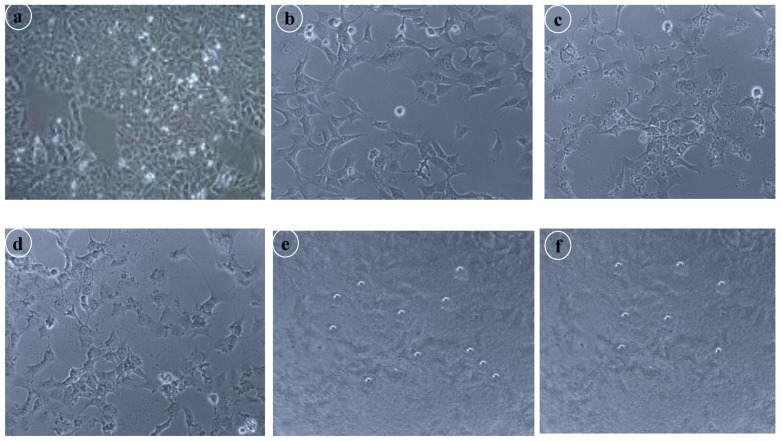
Morphological changes of the human breast cancer cell line MCF-7 after given treatment; (**a**) Control; (**b**) 20 μg/mL of cisplatin-SLNs; (**c**) 40 μg/mL of cisplatin-SLNs; (**d**) 60 μg/mL of cisplatin-SLNs; (**e**) 80 μg/mL of cisplatin-SLNs; (**f**) 100 μg/mL of cisplatin-SLNs.

The cytotoxicity of the prepared formulation was evaluated to establish and confirm the biocompatibility of drug and excipients using an MTT assay, as shown in Figure 6. MCF-7 cell lines have been treated with different concentrations of cisplatin-loaded SLNs to determine the effect on cell proliferation. The results demonstrated that cisplatin-loaded SLNs treatment is effective in repressing cell proliferation. The findings of the study indicated that cell viability ranged from 30% to 40% across all the MCF-7 cell lines. In addition, SLNs loaded with cisplatin had a strong cytotoxic effect only on MCF-7, with an IC_50_ value of 6.51 ± 0.39 μg/mL on the breast cancer cell line, whereas the IC_50_ value of free cisplatin for MCF-7 cells was 10 μg/mL. It also promoted the fragmentation of DNA which was linked to the induction of cell death by apoptosis. The findings showed that in the study of MCF-7 cell lines, the prepared formulation was effective. From Figure 7, it is clear that there was a drastic change in the morphology of the MCF-7 cell lines. It can be concluded that with increasing concentrations of dose, the viability of the cells diminished compared to the standard.

## 4. Conclusions

The clinical success of cisplatin, a well-known anticancer drug, is greatly affected by its nonspecificity and serious dose-limiting toxicities. The microwave-assisted microemulsion method was used in the preparation of SLNs loaded with cisplatin. To improve its ability, we have developed a new stearic acid-functionalized SLNs as cisplatin carrier. The drug-loaded SLNs were within the nanosize range (70.04 nm) and showed a zeta potential of −20 mV, entrapment efficiency of ~70–90%, and loading capacity of 3.6–4.6% (*w/w*), particularly because of the single-pot nature of the microwave method, which allows simultaneous encapsulation of the drug with the formation of SLNs, a high entanglement efficiency, and load capacity. In summary, these results highlighted that cisplatin SLNs enhanced anticancer efficacy in MCF-7 cancer cells.

## Figures and Tables

**Table 1:** Microwave-assisted cisplatin solid lipid nanoparticles for subcutaneous delivery.

Form. Code	CPN * (mg)	Solutol HS 15 (mg)	Tween 80 (mg)	Stearic Acid (mg)	PF68 ^‡^ (mg)	Ascorbic Acid (mg)	Tetraxetan (mg)	Water (mL)
F1	120	50	-	10	75	10	2	1.2
F2	120	50	-	20	150	10	2	1.2
F3	120	50	-	40	225	10	2	1.2
F4	120	50	-	60	300	10	2	1.2
F5	120	50	-	80	400	10	2	1.2
F6	120	-	50	100	75	10	2	1.2
F7	120	-	50	150	150	10	2	1.2
F8	120	-	50	200	225	10	2	1.2
F9	120	-	50	250	300	10	2	1.2
F10	120	-	50	300	400	10	2	1.2

* CPN = Cisplatin, ^‡^ PF68 = Lutrol.
